# Utilizing the electron transfer mechanism of chlorophyll a under light for controlled radical polymerization†
†Electronic supplementary information (ESI) available: Experimental details, Table S1 and Fig. S1–13. See DOI: 10.1039/c4sc03342f


**DOI:** 10.1039/c4sc03342f

**Published:** 2014-11-27

**Authors:** Sivaprakash Shanmugam, Jiangtao Xu, Cyrille Boyer

**Affiliations:** a Centre for Advanced Macromolecular Design and Australian Centre for NanoMedicine , School of Chemical Engineering , The University of New South Wales , Sydney , NSW 2052 , Australia . Email: cboyer@unsw.edu.au ; Email: j.xu@unsw.edu.au ; Fax: +61 2 9385 4749

## Abstract

Efficient photoredox catalysts containing transition metals, such as iridium and ruthenium, to initiate organic reactions and polymerization under visible light have recently emerged. However, these catalysts are composed of rare metals, which limit their applications. In this study, we report an efficient photoinduced living radical polymerization process that involves the use of chlorophyll as the photoredox biocatalyst. We demonstrate that chlorophyll a (the most abundant chlorophyll in plants) can activate a photoinduced electron transfer (PET) process that initiates a reversible addition-fragmentation chain transfer (RAFT) polymerization under blue and red LED light (*λ*_max_ = 461 and 635 nm, respectively). This process controls a wide range of functional and non-functional monomers, and offers excellent control over molecular weights and polydispersities. The end group fidelity was demonstrated by NMR, UV-vis spectroscopy, and successful chain extensions for the preparation of diblock copolymers.

## Abstract 2

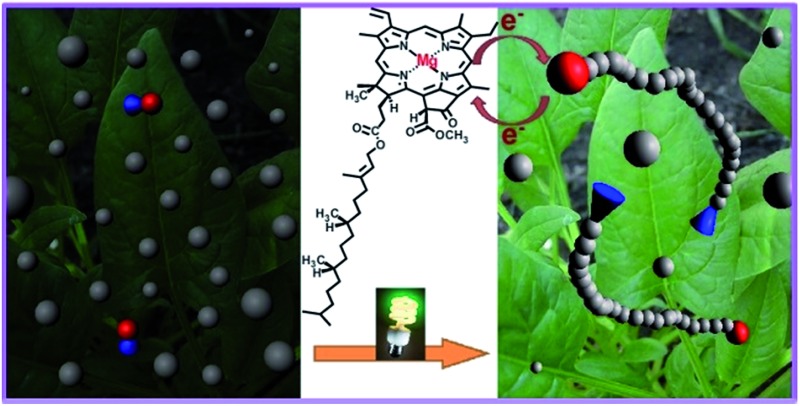
We report an efficient photoinduced living radical polymerization process that involves the use of chlorophyll as the photoredox catalyst, which allows the preparation of well-defined polymers.

## Introduction

The inhabitation of Earth by cyanobacteria approximately 3.4 billion years ago led to the development of an efficient chemical process called photosynthesis. Through photosynthesis, plants, algae and some species of bacteria are able to harvest and convert solar energy to chemical energy to synthesize polysaccharide and natural polymers.^1^ Currently, various research groups have undertaken great efforts to mimic and engineer this sophisticated process through artificial means, which could improve our understanding of the bioenergetics process; lead to the development of more eco-friendly systems, including renewable energy production (solar fuels) and more efficient chemical reactions; and give rise to potential applications in optoelectronics, photonics and sensor design.^2^ In photosynthesis, sunlight is absorbed and converted to electronic excitation energy, which initiates a sequence of photoinduced electron transfer (PET) events and the synthesis of carbohydrates *via* the Calvin–Benson cycle.^3^ Over the past five years, organic chemists, inspired by this process, have developed a technique based on visible light photoredox catalysis to perform synthetic organic transformations.^4^–^9^ This approach relies on the ability of metal complexes and organic dyes to engage in single-electron transfer processes with organic substrates upon photoexcitation with visible light. Additionally, these photosensitizers could be utilized to initiate photopolymerization reactions *via* free radical or cationic mechanisms.^10^–^13^ Recently, Hawker's^14^–^19^ and our research group^20^,^21^ developed new polymerization techniques using photoredox catalysts, such as ruthenium- and iridium-based complexes, to exploit the electrons generated during the PET process and activate/mediate controlled/“living” radical polymerization under visible light. In our work,^20^,^21^ we established a photoinduced electron transfer-reversible addition-fragmentation chain transfer (PET-RAFT) technique that utilizes a PET mechanism to activate thiocarbonylthio compounds to generate radicals and thereby initiate controlled free radical polymerization. Subject to the selection of photocatalysts, *i.e.*, iridium- or ruthenium-based catalysts, a great diversity of monomers were successfully polymerized with excellent control over molecular weights, polydispersities and specific sequences. Although these catalysts are extremely efficient in conducting PET processes for organic transformation or polymerization, they present several deficiencies. First, the catalysts are composed of rare and expensive metals, such as ruthenium and iridium, which limit their potential applications in industry because the metals are found in trace quantities in the Earth's crust (<1 ppm).^22^ Second, ruthenium and iridium complexes are toxic and require several additional purification steps to eliminate any potentially adverse effects they may have in the application of final products.^23^ Therefore, the development of renewable catalysts from bio-resources, capable of conducting the PET process, is highly desirable.

The most abundant natural visible light photocatalyst for PET processes on Earth is chlorophyll, which is the principal photoacceptor in the chloroplasts of most green plants. During photosynthesis, the absorption of a photon excites the chlorophyll from its ground state to its excited state and initiates an electron transfer reaction. This high-energy electron can have several fates. The electron could return to the ground state, with the absorbed energy converted to heat or fluorescence. However, if a suitable electron acceptor with high electron affinity is close to the chlorophyll molecule, the excited electron can be transferred from the initial chlorophyll molecule to the acceptor and generate a positive charge on the chlorophyll molecule (due to the loss of an electron) and a negative charge on the acceptor.^24^ This process is also referred to as photoinduced charge separation. In plants, the electron extracted from chlorophyll is used to reduce species such as water and CO_2_. Despite ongoing research on artificial photosynthesis for solar energy conversion, this is the first example of chlorophyll being used as an efficient photoredox catalyst for the production of high-performance polymeric materials *via* living polymerization. In this study, we discovered that chlorophyll a (Chl a, the most widely distributed form of chlorophyll) could mediate PET-RAFT process and lead to the production of well-defined polymers with controlled molecular weights, polydispersities and end group functionalities.

**Scheme 1:**
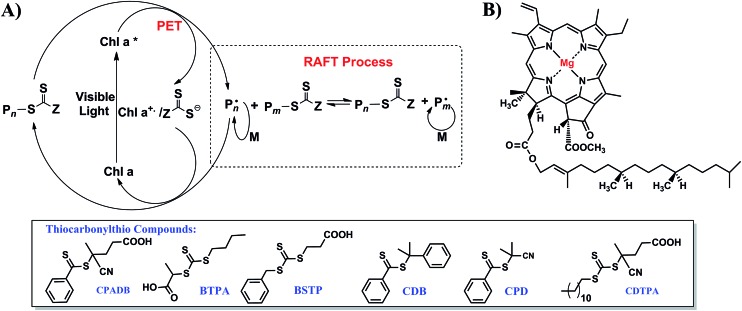
(A) Proposed mechanism for PET-RAFT polymerization with different thiocarbonylthio compounds employing chlorophyll a (Chl a) as biocatalyst and (B) the chemical structure of Chl a.

Because spinach is an affordable and renewable feedstock, it can be used as the raw material for the extraction, isolation and characterization of Chl a.^25^ Chl a was extracted from spinach leaves and purified by column chromatography as previously reported.^25^,^26^ Water miscible solvents such as pyridine, methanol, ethanol, acetone, *N*,*N*-dimethylformamide (DMF) and dimethylsulfoxide (DMSO) are most suitable for extraction of chlorophyll. Extraction in the absence of a suitable solvent may lead to oxidation or hydrolysis of chlorophyll molecules.^27^,^28^ The structure of Chl a and its purity were confirmed by proton nuclear magnetic resonance (^1^H NMR) and UV-vis spectroscopy (ESI, Fig. S2†) and compared to the data reported in the literature.^27^,^29^,^30^ The concentration of Chl a was determined by spectral measurements based on the equation developed by Wellburn.^27^ In our experiments, 24 mg of Chl a was extracted from 100 g of spinach leaves. Inspired by our early work on PET-RAFT polymerization with iridium and ruthenium complexes,^20^,^21^,^31^ we decided to test Chl a as a potential photoredox catalyst to conduct a photocontrolled radical polymerization in the presence of thiocarbonylthio compounds (RAFT agents) (Scheme 1). Chl a is reported to have a half-wave reduction potential of –1.1 V in DMSO *versus* the saturated calomel electrode (SCE) in the excited state.^28^,^32^,^33^ Consequently, Chl a is a strong reducing agent capable of transferring an electron to an oxidant of lower reduction potential to yield a π-cation radical. As the magnesium center in Chl a (Scheme 1B) is a redox-neutral metal, the electron does not originate from the metal center of the Chl a molecule but from the aromatic π-electron system of the porphyrin. This mechanism is in direct contrast with the electron generation mechanism of transition metal photocatalysts (such as ruthenium and iridium) because these photocatalysts rely on metal to ligand charge transfer (MLCT).^20^,^21^ The resultant positive charge of the cationic Chl a and the spin of the unpaired electron are delocalized extensively over the π-electron system.^32^

The theory of reversible one electron oxidation of Chl a to generate π-cation radical has been reported by ferric chloride oxidation, electrolytic oxidation and charge transfer with zinc tetraphenylporphyrin perchlorate (ZnPh_4_P^+^˙).^34^,^35^ In electrolytic oxidation, Chl a underwent one electron oxidation to yield a yellow solution which displayed no strong visible fluorescence.^34^,^36^ Electron paramagnetic resonance (EPR) and absorption studies on this oxidized product proved the presence of π-cation radical of Chl a that could be reduced to regenerate 90% of Chl a upon electroreduction. In addition, π-cation radical Chl a was sufficiently stable to permit its electromigration as a cation to establish its ionic nature through electrophoresis. There is also a possibility for π-cation radical Chl a to form dimers (2Chl^+^˙ = (Chl^+^˙)_2_). However, the formation of dimer is an unlikely pathway as EPR signal of the postulated dimer would require the dimer to be weakly coupled and previous studies on magnesium octaethylporphyrin cation radical displayed no EPR signal. In addition, El Khouly *et al.* have shown that deactivation of π-cation radical Chl a upon electron transfer from ^3^Chl a*/^3^Chl b* to C_60_/C_70_ takes place through diffusion controlled back electron transfer.^24^ Therefore, the π-cation radical species generated through oxidation is capable of extracting an electron from a reducing agent to form the ground state. The reduction of a RAFT agent leads to the generation of a radical (P˙) capable of initiating RAFT polymerization as well as serving as a chain transfer agent. Upon addition of propagating radical (P˙) to the π-cation radical Chl a-thiocarbonylthio complex (Scheme 1A), deactivation of polymerization takes place to yield dormant propagating chain and uncharged Chl a, thereby restarting the catalytic cycle. In another possible but unlikely pathway of deactivation, π-cation radical Chl a-thiocarbonylthio complex directly abstracts an electron from the propagating radical (P˙) to regenerate dormant propagating chain and Chl a. However, the generation of cationic propagating radical will be energetically unfavorable. In addition, there is also a possibility of regenerating Chl a from π-cation radical Chl a-thiocarbonylthio complex through disproportionation of π-cation radical Chl a to Chl a and a di-cation radical Chl a (Chl a^2+^) which can be reduced by nucleophiles and water to form allomers of Chl a.^34^

To confirm that the polymerizations were activated by Chl a and RAFT agent, a range of control experiments was carried out in detail under blue and red light emitting diode (LED) lights. Firstly, the methyl methacrylate (MMA) and methyl acrylate (MA) polymerizations, containing RAFT agents, Chl a and monomers, were performed in the absence of light. In these conditions, no monomer conversion was detected by NMR and gel permeation chromatography (GPC) analysis (data not shown), which demonstrated that the light is required to activate the polymerization. Secondly, the polymerizations were performed in the absence of Chl a or RAFT agents. Upon 10 hours of red light irradiation and 4 ppm of Chl a with respect to monomer concentration in the absence of RAFT agent (2-(*n*-butyltrithiocarbonate)-propionic acid, BTPA), MA showed a negligible conversion to polymer (Table 1, # 2); on the other hand, MMA remained inert even after 13 hours of irradiation (Table 1, # 10). An interesting fact to note was that similar results were achieved for control experiment carried out with MMA in the presence of blue light as no polymerization was observed (ESI, Table S1,† # 2). These results demonstrated that both Chl a and RAFT agents were essential to activate polymerization.

**Table 1:** PET-RAFT Polymerization of a variety of monomers using Chl a as biocatalyst and 4.8 W red LED lamp as a light source (*λ*_max_ = 635 nm)

#	Exp. Cond.^*a*^ [*M*] : [RAFT agent] : [Chl a]	Monomer	RAFT agent	[Chl a]/[*M*] (ppm)	Time (h)	*α* ^*b*^ (%)	*M*_n,th._ ^*c*^ (g mol^–1^)	*M*_n,GPC_ ^*d*^ (g mol^–1^)	*M*_w_/*M*_n_
1	200 : 1 : 8 × 10^–4^	MA	BTPA	4	5	76	13 300	10 800	1.06
2	200 : 0 : 8 × 10^–4^	MA	—	4	10	6	—	—	—
3	200 : 1 : 8 × 10^–4^	MMA	CPADB	4	4	24	5100	6570	1.10
4	200 : 1 : 8 × 10^–4^	MMA	CPADB	4	20	50	10 300	14 650	1.14
5	200 : 1 : 8 × 10^–4^	MMA	CPADB	4	36	94	19 100	20 300	1.13
6	200 : 1 : 2 × 10^–3^	MMA	CPADB	10	25	94	19 100	20 420	1.16
7	200 : 1 : 5 × 10^–3^	MMA	CPADB	25	25	71	14 500	16 700	1.13
8	200 : 1 : 5 × 10^–3^	MMA	CPADB	25	15	50	10 300	12 360	1.15
9	200 : 1 : 5 × 10^–3^	MMA	CPADB	25	10	29	6100	8400	1.12
10	200 : 0 : 8 × 10^–4^	MMA	—	4	20	0	—	—	—
11	200 : 1 : 8 × 10^–4^	NIPAAm	BTPA	4	4	47	10 900	13 970	1.08
12	200 : 1 : 8 × 10^–4^	HPMA	CPADB	4	12	53	15 600	9800 (15 900)^*i*^	1.05
13	200 : 1 : 8 × 10^–4^	HEMA	CPADB	4	6	77	20 330	22 700	1.09
14	200 : 1 : 8 × 10^–4^	PFPA	BTPA	4	6	55	26 180	22 300	1.08
15	200 : 1 : 8 × 10^–4^	GMA	CPADB	4	12	53	15 330	16 300	1.12
16	200 : 1 : 8 × 10^–4^	DMAEMA	CPADB	4	14	20	6300	9600	1.18
17	200 : 1 : 0	DMAEMA	CPADB	0	10	0	—	—	—
18	200 : 1 : 8 × 10^–4^	MA	BSTP	4	3	41	7340	7920	1.20
19	370 : 1 : 8 × 10^–4^	MMA	CDB	4	12	33	12 500	15 550	1.27
20	200 : 1 : 8 × 10^–4^	MMA	CPD	4	12	60	12 240	13 700	1.17
21	200 : 1 : 8 × 10^–4^	MMA	CDTPA	4	14	79	16 200	12 800	1.17
22^*f*^	200 : 1 : 8 × 10^–4^	MA	BTPA	4	8	53	9400	11 500	1.07
23^*g*^	200 : 1 : 8 × 10^–4^	MA	BTPA	4	20	44	7800	8700	1.06
24^*j*^	200 : 1 : 8 × 10^–4^	MMA-*stat*-MAA^*e*^	CPADB	4	9	ND^*h*^	ND^*h*^	25 000	1.19

^*a*^The polymerizations were performed in the absence of oxygen at room temperature in dimethylsulfoxide (DMSO) using 4.8 W red LED lamp as a light source (*λ*_max_ = 635 nm).

^*b*^Monomer conversion was determined by using ^1^H NMR spectroscopy.

^*c*^Theoretical molecular weight was calculated using the following equation: *M*_n,th_ = [*M*]_o_/[RAFT] × MW^M^ × *α* + MW^RAFT^, where [*M*]_o_, [RAFT]_o_, MW^M^, *α*, and MW^RAFT^ correspond to initial monomer concentration, initial RAFT concentration, molar mass of monomer, conversion determined by ^1^H NMR, and molar mass of RAFT agent.

^*d*^Molecular weight and polydispersity were determined by GPC analysis (DMAc as eluent) based on polystyrene standards.

^*e*^[MMA]_0_ : [MAA]_0_ : [RAFT] : [Chl a] = 100 : 100 : 1 : 8 × 10^–4^.

^*f*^The reaction was carried out in *N*,*N*-dimethylformamide (DMF) under red LED light irradiation.

^*g*^The reaction was carried out in acetonitrile (MeCN) under red LED light irradiation.

^*h*^Not determined.

^*i*^Molecular weight determined by ^1^H NMR.

^*j*^Methylation was carried out with trimethylsilyldiazomethane prior to GPC analysis (DMAc eluent) based on polystyrene standards.

Further supporting evidence to the proposed mechanism was obtained through fluorescence quenching studies. The fluorescence spectra of Chl a in DMSO in the presence of varying concentration of RAFT agents, 4-cyanopentanoic acid dithiobenzoate (CPADB) and BTPA, are shown in ESI, Fig. S3 and S4.† The fluorescence of Chl a in DMSO achieves a maximum at 673 nm when excited at 433 nm. Upon addition of RAFT agents (both CPADB and BTPA), progressive quenching of Chl a fluorescence with increasing concentrations of RAFT agents was observed. The decrease of emission is correlated with the Stern–Volmer equation, *I*_0_/*I* = 1 + *k*_q_*τ*_0_[*Q*], where *I*_0_ and *I* are the emission intensity in the absence and presence of quencher, *k*_q_ is the quenching rate constant, *τ*_0_ is the excited lifetime, [*Q*] is the quencher concentration. Plotting *I*_0_/*I* ratio *versus* the concentration of quencher gives a straight line (ESI, Fig. S4†). These results suggest that the excited Chl a engages in single-electron transfer with both BTPA and CPADB.

In contrast to ruthenium and iridium catalysts, Chl a presents two absorption bands in the visible spectrum, *i.e.*, at 430 and 665 nm (ESI, Fig. S2B†), which correspond to the blue (Soret band) and red (Q-band) regions of the visible spectrum, respectively.^27^ It has been demonstrated that both absorption bands induce a PET process during photosynthesis. In our early attempts, we tested the polymerization of MMA and MA under blue (*λ*_max_ = 461 nm) and red LED light (*λ*_max_ = 635 nm) in DMSO. The polymerization of MMA was initially tested using dithiobenzoate (CPADB), whereas that of MA was tested using trithiocarbonate (BTPA). In the presence of RAFT agent and several hours of irradiation with a molar ratio of [monomer] : [RAFT agent] : [Chl a] = 200 : 1 : 8 × 10^–4^, we observed a viscous reaction mixture, which indicated the generation of polymers. The polymerizations proceeded smoothly to high monomer conversions (50% and 76% for MMA (Table 1, # 4) and MA (Table 1, # 1) after 20 h and 5 h of red light irradiation, respectively). The samples were also analyzed by GPC, which revealed the synthesis of well-defined polymers with narrow molecular weight distributions (*M*_w_/*M*_n_ < 1.15) and a good control over molecular weights.

In addition, the polymerization of (meth)acrylamides (Table 1, # 11–12), methacrylates (Table 1, # 13, 15–16), acrylate (Table 1, # 14) and statistical copolymerization of methacrylic acid with methyl methacrylate (Table 1, # 24) were also successfully carried out in the presence of red light and blue light (ESI, Table S1,† # 1, 3–6, and 8) with the synthesis of polymers with narrow molecular weight distributions (*M*_w_/*M*_n_ < 1.25). In the polymerization of DMAEMA, it was found that prolonged irradiation of monomer under blue light in the absence of RAFT agent and catalyst could lead to self-initiation (ESI, Table S1,† # 7). However, no such initiation was reported upon irradiation with red light (Table 1, #17).

In order to further test the versatility of Chl a, we decided to polymerize MA and MMA with RAFT agents other than CPADB and BTPA. Polymerization of MA with 3-benzylsulfanyl-thiocarbonylthiosulfanyl propionic acid (BSTP) was successful (Table 1, # 18) but a little higher polydispersity was observed as compared to that BTPA was used. For MMA, polymerization with 2-cyano-2-propylbenzodithioate (CPD) (Table 1, # 20) and 4-cyano-4-[(dodecylsulfanylthiocarbonyl)sulfanyl] pentanoic acid (CDTPA) (Table 1, # 21) yielded polymers with narrow molecular weight distributions (*M*_w_/*M*_n_ < 1.20); however, polymerization with 2-phenyl-2-propyl benzodithioate (CDB) (Table 1, # 19) yielded a slightly broader molecular weight distribution (*M*_w_/*M*_n_ = 1.27) due to possibly slow initiation of RAFT agent leading to asynchronous chain propagation. Unfortunately, other thiocarbonylthio compounds, such as xanthate (methyl 2-[(ethoxycarbonothioyl) sulfanyl]propanoate) and dithiocarbamate (cyanomethyl methyl-(phenyl)carbamodithioate) investigated for the polymerization of vinyl acetate were unsuccessful (data not shown).

We then tested the tolerance of Chl a with different solvents, including DMF, acetonitrile (MeCN), and toluene. Chl a was effective in polymerizing MA in both DMF (Table 1, # 22) and MeCN (Table 1, # 23) with low polydispersities (*M*_w_/*M*_n_ < 1.10), however, the polymerization in MeCN was much slower. In toluene, no polymerization was observed. In comparing to all the investigated solvents, the strongest ligands for Chl a is DMF and DMSO with donicities of 26.6 and 29.8 kcal mol^–1^ respectively,^37^,^38^ which yields high monomer conversion and good polymerization control. On the other hand, MeCN and toluene have donicities of 19.0 and 0.1 kcal mol^–1^,^37^,^38^ thereby making them poor ligands to solubilize Chl a, which result in a poor control of polymerization.

**Fig. 1:**
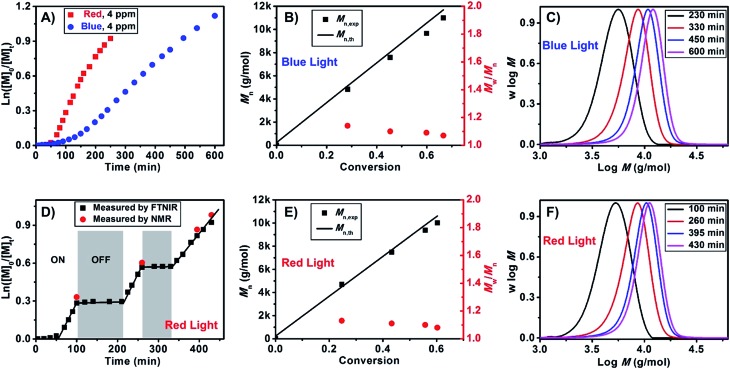
Online Fourier transform near-infrared (FTNIR) measurement for kinetic study of PET-RAFT polymerization of methyl acrylate (MA) at room temperature with Chl a as the photoredox biocatalyst and BTPA as the chain transfer agent under blue (A, B and C) and red (A, D, E and F) light irradiation, using molar ratio of [MA] : [BTPA] : [Chl a] = 200 : 1 : 8 × 10^–4^ in DMSO. (A) Plot of ln([*M*]_0_/[*M*]_*t*_) *vs.* exposure time under blue (blue squares) and red (red dots) lights; (B and E) *M*_n_*vs.* conversion for blue (B) and red (E) light system; (C and F) molecular weight distributions at different time points under blue (C) and red (F) light irradiation; (D) plot of ln([*M*]_0_/[*M*]_*t*_) *vs.* time for conversion of MA in the presence (“ON”) and absence (“OFF”) of red light.

We subsequently investigated the polymerization kinetics using online Fourier transform near-infrared (FTNIR) spectroscopy, which measured the monomer conversions by following the decrease in the vinylic C–H stretching overtone of monomers at ∼6200 cm^–1^, as described in previous publications.^20^ ln([*M*]_0_/[*M*]_*t*_) was plotted against exposure time, as shown in Fig. 1A, to determine the apparent propagation rate constant (*k*appp). Interestingly, a higher propagation rate constant (*k*appp (red) = 5.6 × 10^–3^ min^–1^) and a shorter induction period (50 min) were observed under red light compared to those observed under blue light (*k*appp (blue) = 2.4 × 10^–3^ min^–1^ and 100 min induction period). These findings are contrary to the observed specific absorption coefficient (*α*) for Chl a. Based on previous studies, specific absorption coefficient of Chl a was determined to be 96.6–100.9 at 665 nm (red light) and 125.1–131.5 at 430 nm (blue light).^28^ In other words, polymerization should be faster in blue light than red light. However, these conflicting results can be attributed to the efficiency of Chl a in red light as compared to blue light. Although Chl a is able to absorb high energy blue light, the lowest energy transition only occurs at around 660 nm in solution. This low wavelength transition leads to a change in electron distribution within the porphyrin nuclear framework and eventually electron transfer to a strong electron acceptor.^39^ In the presence of blue light, Chl a is excited to a higher vibrational and electronic singlet states, but this energy is quickly radiated to the environment as random translational energy of heat to reach the first excited singlet state where one of the modes of energy transfer is through oxidation of Chl a.^28^ We propose that the higher activity of Chl a in polymerization of MA lies in its efficiency in absorbing low energy red light which leads to photoinduced electron transfer to BTPA. Moreover, the higher propagation rate for red light as compared to blue light may also come from competitive absorption between RAFT agent and Chl a. RAFT agents such as CPADB and BTPA have a strong absorption peak at 305 nm (and a weak absorption at 520 nm) and exist in excess as compared to Chl a. Upon addition of Chl a, the Soret band at 430 nm of Chl a overlaps with the shoulder of the strong absorption peak of RAFT agents (ESI, Fig. S11A and S11B†). This overlap leads to competitive absorption between Chl a and BTPA/CPADB which may result in lower efficiency of Chl a in blue light as compared to red light. In addition, no other intense light absorption is observed in the visible light spectrum for Chl a. Therefore, polymerization should be observed only in blue and red lights. To test this hypothesis, a polymerization of MA was carried out under green LED light (*λ*_max_ = 530 nm, 4.8 W). As expected, no polymerization was observed under green light, which is attributed to the absence of strong absorbance band. After purification, the presence of thiocarbonylthio end groups in both PMA and PMMA was confirmed by NMR (ESI, Fig. S5 and S6†) and UV-vis spectroscopy (ESI, Fig. S7†). End group fidelity was quantified to be greater than 95% for both polymerizations under blue and red LED light.

Because there are fewer reports^40^–^42^ employing low-energy light (>600 nm, or red light) to activate polymerization than those using high-energy light (<400 nm, blue or UV light),^14^,^43^–^56^ we explored the polymerization of MMA, MA and other monomers under red light in various solvents. Several aliquots were taken at specific intervals during the polymerization of MMA under red light to measure the molecular weights and molecular weight distributions by GPC. By plotting *M*_n_ and polydispersity values against monomer conversion, we observed the characteristics of living radical polymerization, particularly a linear increase in *M*_n_ and a slight decrease in polydispersity (Fig. 1E and F and ESI, Fig. S8B and S8C†) for both MA and MMA. A lower polydispersity was obtained under red light, suggesting better control under red light. An additional feature introduced in this experiment was switching “ON” and “OFF” the light source to demonstrate that Chl a was acting as a molecular switch, which afforded temporal and potentially spatial control. The polymerization of both MA (Fig. 1D) and MMA (ESI, Fig. S8A†) was observed when the light was “ON”. In the absence of light (“OFF”), no monomer conversion was recorded. Aliquots of the reaction mixtures used for MA and MMA polymerization were also taken at specific intervals to measure the molecular weights and molecular weight distributions by GPC and the monomer conversions by NMR analysis. As indicated in Fig. 1D, the conversions at specific times, calculated by FTNIR, were in close agreement with the NMR data.

**Fig. 2:**
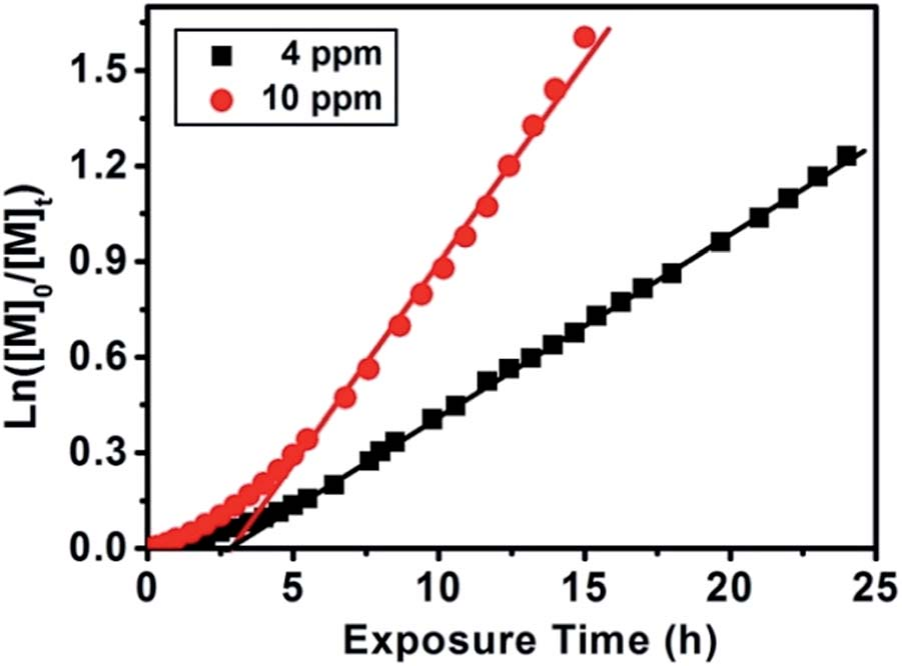
Plotting ln([*M*]_0_/[*M*]_*t*_) against exposure time measured by online FTNIR for different Chl a concentrations (4 ppm against 10 ppm relative to monomer concentration) for the polymerization of MMA at room temperature under red light irradiation with CPADB as chain transfer agent using molar ratio of [MMA] : [CPADB] = 200 : 1 in DMSO. GPC results (*M*_n_*vs.* conversion and molecular weight distributions) showed in Fig. S8† for 4 ppm and Fig. S9† for 10 ppm.

Subsequently, we investigated the effect of Chl a concentration on the polymerization kinetics of MMA *via* on-line FTNIR. The polymerizations were carried out in the presence of 4 ppm and 10 ppm of Chl a relative to the monomer concentration; samples were taken from the reaction mixture at designated times for GPC analysis. By plotting ln([*M*]_0_/[*M*]_*t*_) against time (Fig. 2), we observed linear kinetics that fit the criteria within a first-order approximation for both polymerizations. The propagation rate constants at 10 ppm were determined to be *k*appp (red) = 0.133 h^–1^ and *k*appp (red) = 0.057 h^–1^ at 4 ppm. Consequently, the presence of a higher concentration of catalyst resulted in an increase in the overall rate of polymerization. In addition, the induction period observed in the polymerization of MMA (Fig. 2) and MA (Fig. 1A) can be attributed to stable and long lifetime intermediate of radical addition product in the RAFT process, which was observed in conventional RAFT polymerization and proved by other research groups.^57^–^64^ Analysis of aliquots obtained throughout the course of the polymerization showed a linear increase in molecular weight as a function of conversion. However, a decrease in polydispersity was only observed for the 4 ppm catalyst concentration and not for the 10 ppm concentration (ESI, Fig. S8B & C and S9B & C,† respectively). The higher molecular weight distribution in 10 ppm as compared to 4 ppm is due to termination from oxygen contamination introduced by frequent sampling during the reaction. A repetition of these experiments with no sampling during the course of reaction revealed that at conversions 94% for both 10 ppm (Table 1, # 6) and 4 ppm (Table 1, # 5) Chl a concentrations (relative to monomer concentration), the molecular weight distributions of the homopolymers remain low (PDI < 1.20). Surprisingly, an increase in catalyst concentration from 4 ppm to 10 ppm led to a higher propagation rate constant with negligible changes to the molecular weight and molecular weight distributions (in an inert environment) even at high monomer conversions (>90%) (ESI, Fig. S10†). As both 4 ppm and 10 ppm Chl a show a similar trend at high monomer conversions, we attempted to further increase the concentration of Chl a to 25 ppm (Table 1, # 7–9) to determine the validity of this trend. Interestingly, Chl a concentrations of 4 ppm (Fig. 2) and 25 ppm (Table 1, # 7) have similar polymerization rates by comparing the polymerization of MMA at roughly 70% monomer conversion while the polymerization at 10 ppm (Table 1, # 6) is much faster than that at 25 ppm. The lower polymerization rate for 25 ppm compared to 10 ppm of Chl a is related to self-quenching of Chl solutions at higher concentrations. The mechanism of concentration quenching relies on transfer of excitation energy to statistical pairs of Chl a, which are separated by small distance in solutions, acting as quenching sites. At low concentration of Chl a solutions, fluorescence intensity is independent of concentration; however, at higher concentrations, fluorescence intensity decreases as there is rapid transport of excitonic energy to quenching sites. In the presence of these quenching sites, reduction of RAFT agent through photoinduced electron transfer competes with energy transfer to statistical pairs of Chl a molecules leading to observation of a slower rate for 25 ppm of Chl a as compared to 4 ppm of Chl a.^65^–^71^

**Fig. 3:**
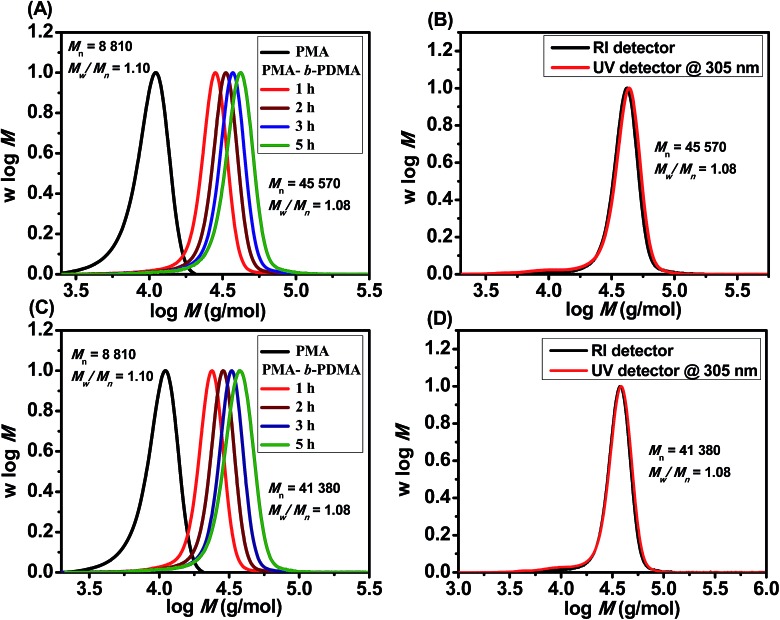
Molecular weight distributions of PMA macroinitiators and their diblock copolymers prepared at room temperature in the presence of Chl a and BTPA as chain transfer in DMSO: (A) molecular weight distributions of PMA macroinitiator and PMA-*b*-PDMA diblock copolymers at 1, 2, 3, and 5 h prepared under red light irradiation; (B) overlap of UV and RI GPC traces of PMA-*b*-PDMA diblock copolymer obtained at 5 h from (A); (C) molecular weight distributions of PMA macroinitiator and PMA-*b*-PDMA diblock copolymers at 1, 2, 3 and 5 h prepared under blue light irradiation; and (D) overlap of UV and RI GPC traces of PMA-*b*-PDMA diblock copolymer obtained at 5 h from (C).

The livingness of the polymers synthesized by PET-RAFT using Chl a was further investigated by chain extensions of PMA and PMMA under both blue and red light. PMA macroinitiators were first synthesized in DMSO under irradiation by blue and red light (*M*_n,GPC_ = 8810 g mol^–1^, *M*_w_/*M*_n_ = 1.10 and 46% monomer conversion for both lights) with BTPA in the presence of 4 ppm Chl a for 3 and 2 h, respectively. A molar ratio of 500 : 1 of the monomer *N*,*N*-dimethylacrylamide (DMA) to the PMA macroinitiator was then used for chain extension in the presence of 4 ppm of Chl a. Successful chain extension was observed for both macroinitiators under blue and red light (Fig. 3A and C), with the molecular weight distributions showing a complete shift in both macroinitiators to higher molecular weights over time. In addition, the UV and RI curves for the diblock copolymers under red and blue lights at 5 h (Fig. 3B and D), show a perfect overlap with the absence of dead chains and a decrease in polydispersities (PMA-*b*-PDMA : *M*_n,GPC,red_ = 45 570 g mol^–1^, *M*_w_/*M*_n_ = 1.08 and 79% monomer conversion for red light, and *M*_n,GPC,blue_ = 41 380 g mol^–1^, *M*_w_/*M*_n_ = 1.08 and 69% monomer conversion for blue light). Successful chain extension of the PMMA macroinitiators with *tert*-butyl methacrylate (*t*BuMA) (ESI, Fig. S12†) and oligo(ethylene glycol) methyl ether methacrylate (OEGMA) (ESI, Fig. S13†) monomers with a molar ratio of [monomer] : [macroinitiator] = 500 : 1 was also demonstrated by GPC.

**Fig. 4:**
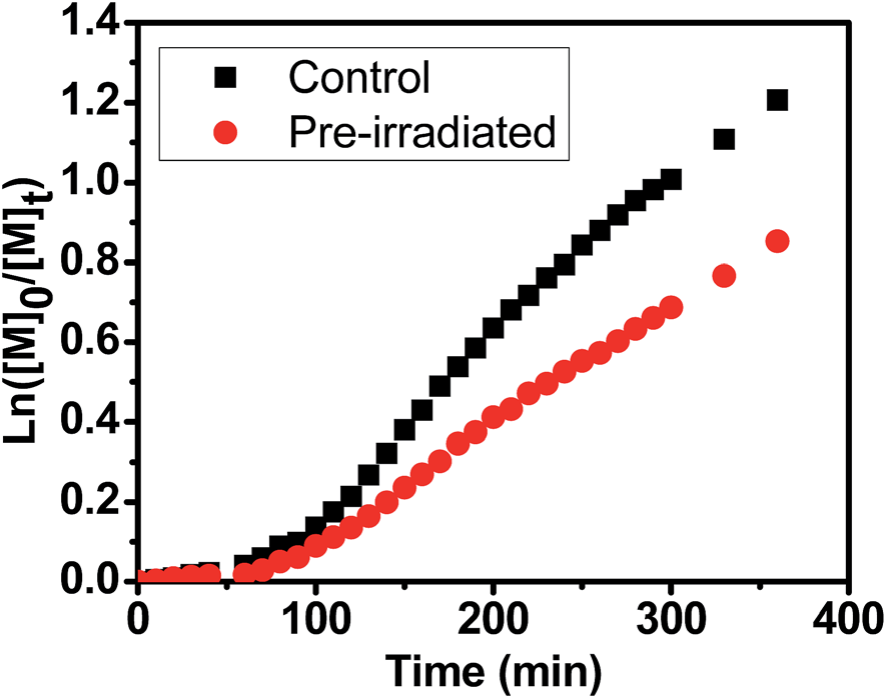
Kinetic study with online Fourier transform near-infrared (FTNIR) measurement for the polymerization of methyl acrylate (MA) in the presence and absence of irradiation under red light with Chl a as the photoredox biocatalyst and BTPA as the chain transfer using molar ratio of [MA] : [BTPA] : [Chl a] = 200 : 1 : 8 × 10^–4^ in DMSO.

In order to investigate the stability of chlorophyll molecule in PET-RAFT polymerization upon prolonged exposure to light, catalyst photostability test was carried out with online FTNIR measurement similar as that we reported on ruthenium before.^72^ For this investigation, two DMSO solutions in two quartz cuvettes containing the same concentration of Chl a (4 ppm) were both degassed with nitrogen. The first cuvette was pre-irradiated under red light for 16 hours, while the second was kept in the dark as a parallel control. Both of them were then employed for the polymerization of MA in the presence of BTPA with a molar ratio of [MA] : [BTPA] : [Chl a] = 200 : 1 : 8 × 10^–4^. The online FTNIR study showed that the polymerization of MA (Fig. 4) in control system (*k*appp (control) = 5.23 × 10^–3^ min^–1^) was faster than that in pre-irradiated one (*k*appp (pre-irradiated) = 3.54 × 10^–3^ min^–1^), indicating of partial degradation of Chl a during light irradiation. This is possibly attributed to the formation of a tetrapyrrole structure through the cleavage of the porphyrin ring at one of the methine bridges.^73^

## Conclusion

In this study, we demonstrated, for the first time, the use of Chl a to mediate a living radical polymerization under blue and red LED light *via* photoinduced electron transfer – reversible addition fragmentation chain transfer (PET-RAFT) polymerization. This polymerization requires only ppm levels of Chl a to activate the PET-RAFT process. Because iridium- and ruthenium-based catalysts are expensive and potentially toxic, this study represented a significant step towards the development of new sustainable and non-transition metal catalysts from bio-resources. A wide range of monomer families, including (meth)acrylamide and (meth)acrylates containing a large variety of functional groups, such as carboxylic acid, amine, alcohol, and glycidyl groups, was successfully polymerized within a few hours and showed excellent control over molecular weight and polydispersity.

## Supplementary Material

Supplementary information

## References

[cit1] Atkins P. W. and Atkins P. W., Elements of physical chemistry, Oxford University Press, Oxford, 6th edn, 2013.

[cit2] Ciamician G. (1912). Science.

[cit3] Bassham J. A., Benson A. A., Calvin M. (1950). J. Biol. Chem..

[cit4] Nicewicz D. A., MacMillan D. W. C. (2008). Science.

[cit5] Narayanam J. M. R., Stephenson C. R. J. (2011). Chem. Soc. Rev..

[cit6] Tucker J. W., Stephenson C. R. J. (2012). J. Org. Chem..

[cit7] Yoon T. P., Ischay M. A., Du J. (2010). Nat. Chem..

[cit8] Prier C. K., Rankic D. A., MacMillan D. W. C. (2013). Chem. Rev..

[cit9] Roy D., Sumerlin B. S. (2014). Macromol. Rapid Commun..

[cit10] Alfredo N. V., Jalapa N. E., Morales S. L., Ryabov A. D., Le Lagadec R., Alexandrova L. (2012). Macromolecules.

[cit11] Kalyanasundaram K. (1982). Coord. Chem. Rev..

[cit12] Lalevée J., Blanchard N., Tehfe M.-A., Peter M., Morlet-Savary F., Fouassier J. (2012). Polym. Bull..

[cit13] Versace D.-L., Cerezo Bastida J., Lorenzini C., Cachet-Vivier C., Renard E., Langlois V., Malval J.-P., Fouassier J.-P., Lalevée J. (2013). Macromolecules.

[cit14] Fors B. P., Hawker C. J. (2012). Angew. Chem., Int. Ed..

[cit15] Fors B. P., Poelma J. E., Menyo M. S., Robb M. J., Spokoyny D. M., Kramer J. W., Waite J. H., Hawker C. J. (2013). J. Am. Chem. Soc..

[cit16] Leibfarth F. A., Mattson K. M., Fors B. P., Collins H. A., Hawker C. J. (2013). Angew. Chem., Int. Ed..

[cit17] Poelma J. E., Fors B. P., Meyers G. F., Kramer J. W., Hawker C. J. (2013). Angew. Chem., Int. Ed..

[cit18] Treat N. J., Sprafke H., Kramer J. W., Clark P. G., Barton B. E., Read de Alaniz J., Fors B. P., Hawker C. J. (2014). J. Am. Chem. Soc..

[cit19] Treat N. J., Fors B. P., Kramer J. W., Christianson M., Chiu C.-Y., Alaniz J. R. d., Hawker C. J. (2014). ACS Macro Lett..

[cit20] Shanmugam S., Xu J., Boyer C. (2014). Macromolecules.

[cit21] Xu J., Jung K., Atme A., Shanmugam S., Boyer C. (2014). J. Am. Chem. Soc..

[cit22] Schroder K., Matyjaszewski K., Noonan K. J. T., Mathers R. T., Simakova A., Mackenzie M., Averick S., Park S., Matyjaszewski K. (2014). Green Chem..

[cit23] Allardyce C. S., Dyson P. J. (2001). Platinum Met. Rev..

[cit24] El-Khouly M. E., Araki Y., Fujitsuka M., Watanabe A., Ito O. (2001). Photochem. Photobiol..

[cit25] Johnston A., Scaggs J., Mallory C., Haskett A., Warner D., Brown E., Hammond K., McCormick M. M., McDougal O. M. (2013). J. Chem. Educ..

[cit26] Quach H. T., Steeper R. L., Griffin G. W. (2004). J. Chem. Educ..

[cit27] Wellburn A. R. (1994). J. Plant Physiol..

[cit28] Vernon L. P. and Seely G. R., The chlorophylls, Academic Press, New York, 1966.

[cit29] Smith K. M., Goff D. A., Abraham R. J. (1984). Org. Magn. Reson..

[cit30] Abraham R. J. and Rowan A. E., in Chlorophylls, ed. H. Scheer, CRC Press, Boca Raton, FL, 1991,.pp. 797–834.

[cit31] Fu C., Xu J., Tao L., Boyer C. (2014). ACS Macro Lett..

[cit32] Zubay G. L. and Geoffrey L. Z., Biochemistry, W. M. C. Brown Publishers, Dubuque, Iowa, 3rd edn, 1993.

[cit33] Mortimer R. G., in Physical Chemistry, Academic Press, Burlington, 2nd edn, 2000, pp. 751–815.

[cit34] Borg D. C., Fajer J., Felton R. H., Dolphin D. (1970). Proc. Natl. Acad. Sci. U. S. A..

[cit35] Dolphin D., Felton R. H. (1974). Acc. Chem. Res..

[cit36] Fuhrhop J. H., Mauzerall D. (1969). J. Am. Chem. Soc..

[cit37] Agostiano A., Cosma P., Trotta M., Monsù-Scolaro L., Micali N. (2002). J. Phys. Chem. B.

[cit38] Gutmann V. (1976). Coord. Chem. Rev..

[cit39] Govindjee A. J. and Govindjee R., Bioenergetics of photosynthesis, Academic Press, New York, 1974.

[cit40] Tehfe M., Louradour F., Lalevée J., Fouassier J.-P. (2013). Appl. Sci..

[cit41] Xiao P., Dumur F., Graff B., Fouassier J. P., Gigmes D., Lalevée J. (2013). Macromolecules.

[cit42] Xiao P., Dumur F., Zhang J., Fouassier J. P., Gigmes D., Lalevée J. (2014). Macromolecules.

[cit43] Tasdelen M. A., Ciftci M., Yagci Y. (2012). Macromol. Chem. Phys..

[cit44] Ciftci M., Tasdelen M. A., Yagci Y. (2014). Polym. Chem..

[cit45] Ribelli T. G., Konkolewicz D., Bernhard S., Matyjaszewski K. (2014). J. Am. Chem. Soc..

[cit46] Zhao Y., Yu M., Zhang S., Liu Y., Fu X. (2014). Macromolecules.

[cit47] Ribelli T. G., Konkolewicz D., Pan X., Matyjaszewski K. (2014). Macromolecules.

[cit48] Anastasaki A., Nikolaou V., Simula A., Godfrey J., Li M., Nurumbetov G., Wilson P., Haddleton D. M. (2014). Macromolecules.

[cit49] Anastasaki A., Nikolaou V., Pappas G. S., Zhang Q., Wan C., Wilson P., Davis T. P., Whittaker M. R., Haddleton D. M. (2014). Chem. Sci..

[cit50] Anastasaki A., Nikolaou V., Zhang Q., Burns J., Samanta S. R., Waldron C., Haddleton A. J., McHale R., Fox D., Percec V., Wilson P., Haddleton D. M. (2014). J. Am. Chem. Soc..

[cit51] Xu J., Atme A., Marques Martins A. F., Jung K., Boyer C. (2014). Polym. Chem..

[cit52] Wolpers A., Vana P. (2014). Macromolecules.

[cit53] Lalevée J., Peter M., Dumur F., Gigmes D., Blanchard N., Tehfe M.-A., Morlet-Savary F., Fouassier J. P. (2011). Chem.–Eur. J..

[cit54] Lalevée J., Tehfe M.-A., Dumur F., Gigmes D., Blanchard N., Morlet-Savary F., Fouassier J. P. (2012). ACS Macro Lett..

[cit55] Lalevée J., Telitel S., Xiao P., Lepeltier M., Dumur F., Morlet-Savary F., Gigmes D., Fouassier J.-P. (2014). Beilstein J. Org. Chem..

[cit56] Konkolewicz D., Schröder K., Buback J., Bernhard S., Matyjaszewski K. (2012). ACS Macro Lett..

[cit57] Wong L., Boyer C., Jia Z., Zareie H. M., Davis T. P., Bulmus V. (2008). Biomacromolecules.

[cit58] Han X., Fan J., He J., Xu J., Fan D., Yang Y. (2007). Macromolecules.

[cit59] Benaglia M., Rizzardo E., Alberti A., Guerra M. (2005). Macromolecules.

[cit60] Perrier S., Barner-Kowollik C., Quinn J. F., Vana P., Davis T. P. (2002). Macromolecules.

[cit61] Barner-Kowollik C., Buback M., Charleux B., Coote M. L., Drache M., Fukuda T., Goto A., Klumperman B., Lowe A. B., McLeary J. B., Moad G., Monteiro M. J., Sanderson R. D., Tonge M. P., Vana P. (2006). J. Polym. Sci., Part A: Polym. Chem..

[cit62] Klumperman B., van den Dungen E. T. A., Heuts J. P. A., Monteiro M. J. (2010). Macromol. Rapid Commun..

[cit63] Konkolewicz D., Hawkett B. S., Gray-Weale A., Perrier S. (2008). Macromolecules.

[cit64] Ranieri K., Delaittre G., Barner-Kowollik C., Junkers T. (2014). Macromol. Rapid Commun..

[cit65] Shi W.-J., Barber J., Zhao Y. (2013). J. Phys. Chem. B.

[cit66] Watson W. F., Livingston R. (1950). J. Chem. Phys..

[cit67] Tweet A. G., Bellamy W. D., Gaines G. L. (1964). J. Chem. Phys..

[cit68] Beddard G. S., Carlin S. E., Porter G. (1976). Chem. Phys. Lett..

[cit69] Agrawal M. L., Chauvet J. P., Patterson L. K. (1985). J. Phys. Chem..

[cit70] Beddard G. S., Porter G. (1976). Nature.

[cit71] Knox R. S. (1994). J. Phys. Chem..

[cit72] Xu J., Jung K., Boyer C. (2014). Macromolecules.

[cit73] Jones C. E., Mackay R. A. (1978). J. Phys. Chem..

