# Supplementary material for: Utilizing the electron transfer mechanism of chlorophyll a under light for controlled radical polymerization
Source: Chem Sci. 2014 Nov 27;6(2):1341–9. doi: 10.1039/c4sc03342f (PMC5811133; doi:10.1039/c4sc03342f)
Supplement: Supplementary file 1 [file SC-006-C4SC03342F-s001.pdf]

Electronic Supplementary Material (ESI) for Chemical Science.
This journal is © The Royal Society of Chemistry 2014

Electronic Supporting Information

Utilizing the Electron Transfer Mechanism of Chlorophyll A under Light for Controlled Radical Polymerization

Sivaprakash Shanmugam[a], Jiangtao Xu[a,b], and Cyrille Boyer[a,b]**

a- Centre for Advanced Macromolecular Design (CAMD), School of Chemical Engineering, UNSW Australia, Sydney, NSW 2052, Australia

b- Australian Centre for NanoMedicine, School of Chemical Engineering, UNSW Australia, Sydney, NSW 2052, Australia

Experimental Section

Materials: Methyl methacrylate (MMA, 99%), *tert*-butyl methacrylate (*t*BuMA, 99%), methyl acrylate (MA, 99%), oligo (ethylene glycol) methyl ether methacrylate (OEGMA, average M_n 300), *N,N*-dimethylacrylamide (DMA, 99%), *N*-isopropylacrylamide (NIPAAm, 97%), glycidyl methacrylate (GMA, 97%), pentafluorophenyl acrylate (PFPA, 98%), methacrylic acid (MAA, 99%), 2-(dimethylamino)ethyl methacrylate (DMAEMA, 98%), *N*-(2-hydroxypropyl) methacrylamide (HPMA, Polysciences Inc., 97%), 2-phenyl-2-propyl benzodithioate (CDB, 99%), 4-Cyano-4-[(dodecylsulfanylthiocarbonyl)sulfanyl]pentanoic acid (CDTPA, 97 %), and 2-cyano-2-propylbenzodithioate (CPD, >97%) were all purchased from Aldrich. Monomers were deinhibited by percolating over a basic alumina column (Ajax Chemical, AR). *N,N*'-dimethylformamide (DMF, 99.8 %, Ajax Chemical), dimethyl sulphoxide (DMSO, Ajax Chemical), diethyl ether (Ajax Chemical), petroleum spirit (Ajax Chemical), *n*-hexane (Ajax Chemical), acetonitrile (Ajax Chemical), and toluene (Ajax Chemical) were used as received. Chlorophyll a (Chl a) was extracted from spinach leaves with acetone and isolated with column chromatography using hexane and acetone mixtures in an alumina column.[1] The structure of Chl a and its purity was confirmed by NMR (**Bruker Avance III 500**) and UV-vis spectroscopy (SI, Figure S1) and compared to the data in the literature. The concentration of Chl a was determined in DMSO by spectral measurements based on the equation developed by Wellburn.[2] Thiocarbonylthiol compounds: 4-cyanopentanoic acid dithiobenzoate (CPADB), 2-(n-

butyltrithiocarbonate)-propionic acid (BTPA) and 3-benzylsulfanylthiocarbonylthiosulfanyl propionic acid (BSTP) were synthesized according to literature procedures.[4,5]

Instrumentation

Gel Permeation Chromatography (GPC) was carried out on synthesized polymer with dimethylacetamide (DMAc) as the eluent. The GPC instrument consists of Shimadzu modular system with an autoinjector, a Phenomenex 5.0 μM bead sizeguard column (50 x 7.5 mm) followed by four Phenomenex 5.0 μM bead size columns (10^5, 10^4 , 10^3 and 10^2 Å) for DMAc system, and a differential refractive-index detector and a UV detector (λ = 305 nm). The DMAc GPC system was calibrated based on narrow molecular weight distribution of polystyrene standards with molecular weights of 200 to 10^6 g mol^{-1}.

Nuclear Magnetic Resonance (NMR) spectroscopy was carried out with Bruker Avance III with SampleXpress operating at 300 MHz for ^{1}H using $CDCl_3$ as solvent and **Bruker Avance III 500 operating at 500** MHz for ^{1}H using acetone-d6 as solvent**.** Tetramethylsilane (TMS) was used as a reference. The data obtained was reported as chemical shift (*δ*) measured in ppm downfield from TMS.

On-line Fourier Transform Near-Infrared (FTNIR) spectroscopy was used for determination of monomer conversion by mapping the decrement of the vinylic C-H stretching overtone of the monomer at ~ 6200 cm^{-1}. A Bruker IFS 66/S Fourier transform spectrometer equipped with a tungsten halogen lamp, a CaF_2 beam splitter and liquid nitrogen cooled InSb detector was used. Polymerizations in blue or red LED lights were carried out using FT-NIR quartz cuvette (1 cm × 2 mm). A spectrum composed of 16 scan with a resolution of 4 cm^{-1} was collected in the spectral region between 7000-4000 cm^{-1} by manually placing the sample into the holder at time intervals of 5, 10, or 30 minutes. The total collection time per spectrum was about 10 seconds and analysis was carried out with OPUS software.

UV-vis Spectroscopy spectra were recorded using a CARY 300 spectrophotometer (Varian) equipped with a temperature controller.

Fluorescence spectroscopy. Fluorescence spectra were recorded using Agilent fluorescent spectrometer.

Photopolymerization was carried out in the reaction vessel where the reaction mixtures are irradiated by

RS Component PACK LAMP RGB blue/red LED lights (4.8 W, $_{max}$ = 461 nm (blue) and 635 nm

(red)) shown below. The distance of the samples to light bulb was 6 cm. The RGB multi-coloured LED light bulb with remote control was purchased from RS Components Australia.

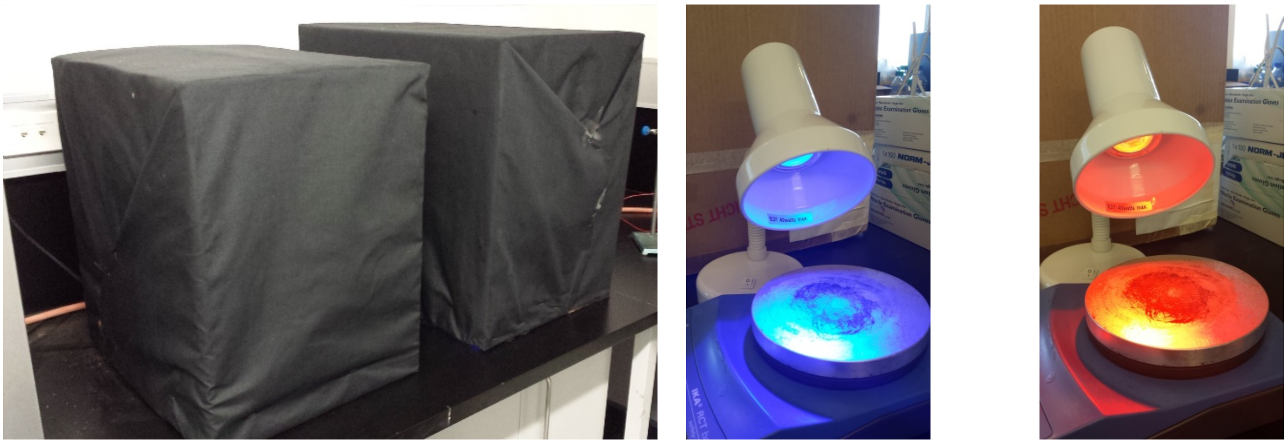

Figure S1. Reaction setup for photopolymerization in this study.

General Procedure for the Synthesis of Methyl Acrylate (MA) via PET-RAFT Polymerization.

Polymerization of MA was carried out in a 5mL glass vial with a rubber septum in the presence of DMSO (370 µL), MA (0.361 g, 4.19 mmol), BTPA (5 mg, 20.97 µmol), and Chl a (75 µL of 224 µM of Chl a stock solution, 0.017 µmol). The glass vial was wrapped with aluminium foil and degassed with nitrogen for 30 minutes. The degassed mixture was then irradiated in red LED light (4.8 W, $_{max}$ = 635 nm (red)) at room temperature. After 5 hours of irradiation, the reaction mixture was removed from the light source in order to be analysed by ^{1}H NMR ($CDCl_3$) and GPC (DMAc) to determine the conversions, number-average molecular weights (M_n) and polydispersities (M_w/M_n).

General Procedures for Kinetic Studies of PET-RAFT Polymerization of Methyl Methacrylate (MMA) with Online Fourier Transform Near-Infrared (FTNIR) Spectroscopy.

A reaction stock solution consisting of DMSO (294 µL), MMA (0.358 g, 3.58 mmol), CPADB (5 mg, 17.90 µmol), and Chl a (64 µL of 224 µM of Chl a stock solution, 0.017 µmol) was prepared in a glass vial. Approximately 500 µL of stock solution was transferred into a 0.9 mL FTNIR quartz cuvette (1 cm × 2 mm) covered with aluminium foil. The reaction mixture in the cuvette was degassed for 30 minutes with nitrogen. The quartz cuvette was then irradiated in red LED light (4.8 W, $_{max}$ = 635 nm (red)) at room temperature. The cuvette was transferred to a sample holder manually for FTNIR measurements every 20 minutes. After 15 seconds of scanning, the cuvette was transferred back to the irradiation source. Monomer conversions were calculated by taking the ratio of integrations of the wavenumber area 6250-6150 cm^{-1} for all curves at different reaction times to that of 0 minutes. Aliquots of reaction samples were

taken at specific time points during the reaction to be analysed by ^{1}H NMR ($CDCl_3$) and GPC (DMAc) to determine the conversions, number average molecular weights (M_n) and polydispersities (M_w/M_n).

General Procedures for Preparation of PMA-*b*-PDMA Diblock Copolymers by PET-RAFT.

In the synthesis of PMA-*b*-PDMA diblock copolymers, MA was polymerized in a 5mL glass vial containing DMSO (740 μL), MA (0.722 g, 8.38 mmol), BTPA (10 mg, 41.94 μmol), and Chl a (150 μL of 224 μM of Chl a stock solution, 0.034 μmol) sealed with a rubber septum. The reaction mixture was then covered with aluminium foil and degassed for 30 minutes with nitrogen. The reaction mixture was irradiated in red LED light (4.8 W, $_{max}$ = 635 nm (red)) at room temperature for 2 hours. The final reaction mixture was purified by precipitating in a mixture of methanol/petroleum spirit (1/1, v/v) with stirring. The pale yellow precipitate was collected and redissolved in minimum amount of dichloromethane before precipitating a second time in methanol/petroleum spirit (1/1, v/v) mixture. The precipitate was analysed in GPC and ^{1}H NMR: $M_{n,GPC}$ = 8 810 g/mol, M_w/M_n = 1.10 and 46 % monomer conversion.

Chain extension of PMMA macroinitiator to DMA was carried out in a 5 mL glass vial in the presence of DMSO (495 μL), DMA (0.366 g, 3.69 mmol), PMMA macroinitiator (0.065 g, 7.38 μmol), and Chl a (66 μL of 224 μM of Chl a stock solution, 0.015 μmol) sealed with a rubber septum. Aluminium foil was used to cover the reaction mixture before degassing for 30 minutes with nitrogen. The reaction mixture was irradiated in red LED light (4.8 W, $_{max}$ = 635 nm (red)) at room temperature for 5 hours. The final reaction mixture was purified by precipitating in a mixture of methanol/petroleum spirit (1/1, v/v) with stirring. The pale yellow precipitate was collected and redissolved in minimum amount of dichloromethane before precipitating a second time in methanol/petroleum spirit (1/1, v/v) mixture. The precipitate was analysed in GPC and ^{1}H NMR: $M_{n,GPC}$ = 45 570 g/mol, M_w/M_n = 1.08 and 79 % monomer conversion. RAFT end group fidelity was determined by using UV-Vis spectroscopy.

The photostability test of Chl a.

A reaction stock solution consisting of DMSO (370 μL) and Chl a (75 μL of 224 μM of Chl a stock solution, 0.017 μmol) was prepared in a 0.9 mL FTNIR quartz cuvette (1 cm × 2 mm) covered with aluminium foil. The reaction mixture in the cuvette was degassed for 30 minutes with nitrogen. The quartz cuvette was then irradiated in red LED light (4.8 W, λ_{max} = 635 nm (red)) at room temperature for 16 h. Another quartz cuvette containing the same formulation was degassed for 30 min with nitrogen, and then was kept in the dark as a parallel control.

After 16 h, MA (0.361 g, 4.19 mmol) and BTPA (5 mg, 20.97 μmol) was added into both cuvettes and sealed with rubber septa. The final reaction mixtures were degassed for 30 min with nitrogen. The cuvette was then irradiated under red light at room temperature. The monomer conversions were monitored by online FTNIR spectroscopy.

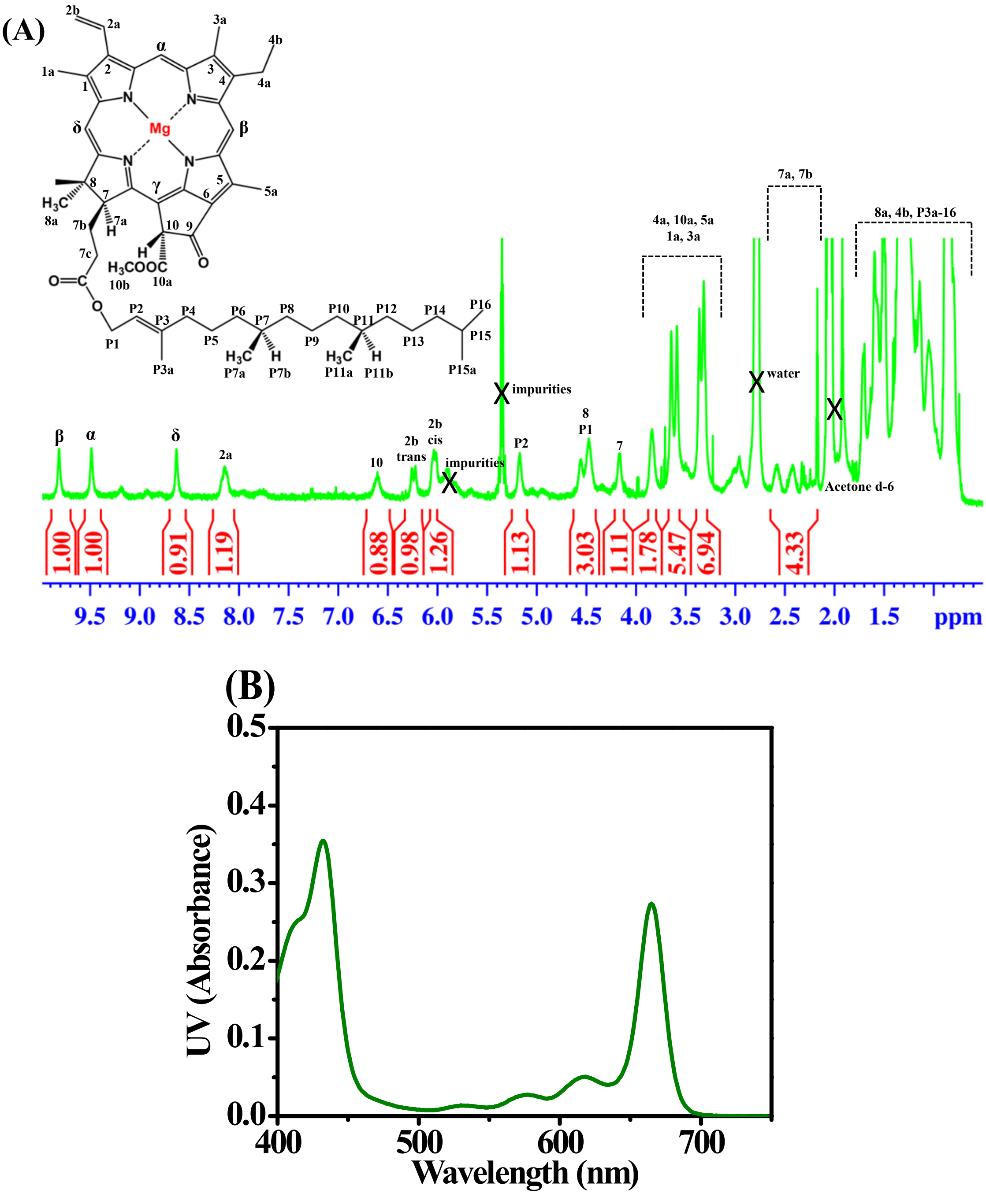

Figure S2. Characterization of purified chlorophyll a by ^{1}H NMR (A) and UV-vis spectroscopy (B) based on previous literature.[3]

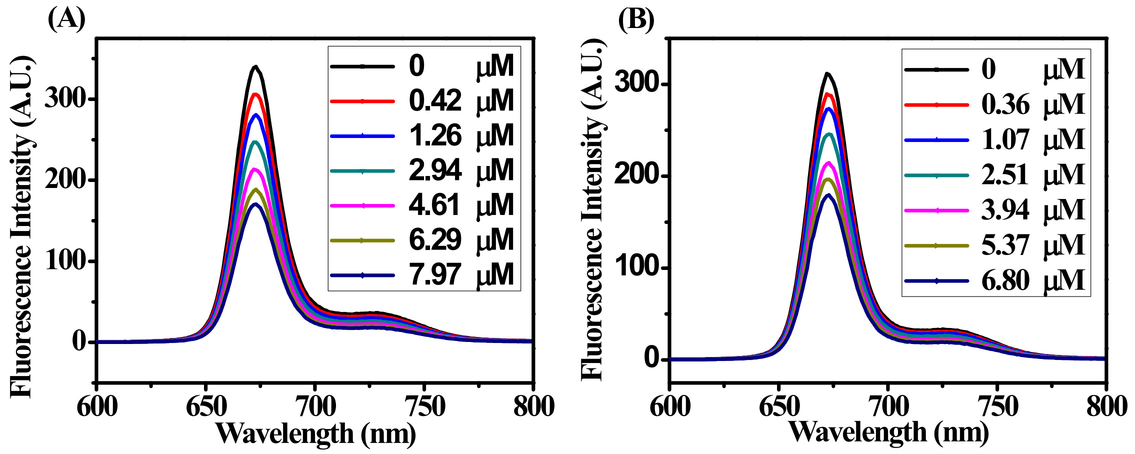

Figure S3. Fluorescence emmission spectra of 0.134 μM Chl a in DMSO in the presence of different concentrations of BTPA (A) and CPADB (B).

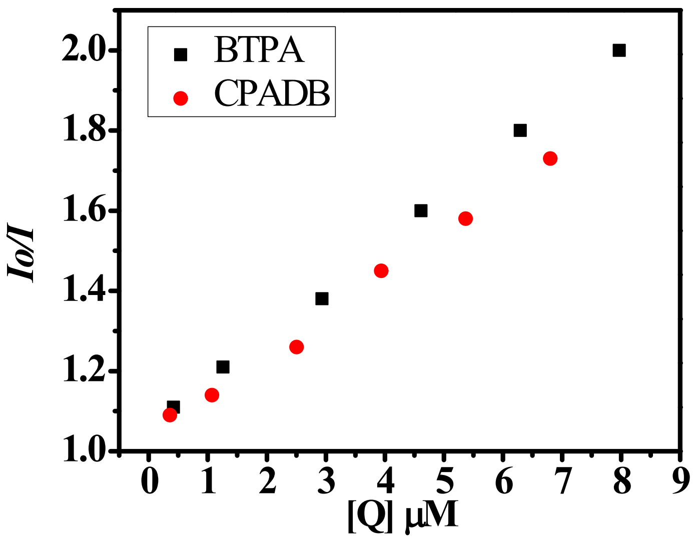

Figure S4. Stern-Volmer plot of Chl a quenching by BTPA and CPADB where I_0, I and [Q] represent emission of Chl a in the absence of quencher (RAFT agents), emission of Chl a in the presence of RAFT agents, and concentration of RAFT agents, respectively.

Table S1. Polymerization of a variety of monomers by PET-RAFT using Chl a as biocatalyst and 4.8 W blue LED lamp as a light source.

#	Exp. Cond.[a] [M]:[CTA]:[Chl a]	Monomer	RAFT agent	[Chl a]/[M] (ppm)	Time (h)	α [b] (%)	$M_{n, th.}$ [c] (g/mol)	$M_{n,GPC}$ [d] (g/mol)	M_w/M_n [d]
1	200:1:8 × 10^{-4}	MMA	CPADB	4	10	21	5 100	4 500	1.11
2	200:0:8 × 10^{-4}	MMA	-	4	13	0	-	-	-
3	200:1:8 × 10^{-4}	NIPAAm	BTPA	4	4	48	11 100	14 530	1.09
4	200:1:8 × 10^{-4}	HEMA	CPADB	4	6	77	20 330	22 800	1.09
5	200:1:8 × 10^{-4}	GMA	CPADB	4	13	47	13 630	14 660	1.14
6	200:1:8 × 10^{-4}	DMAEMA	CPADB	4	14	35	11 300	13 400	1.14
7	200:1:0	DMAEMA	CPADB	4	14	7	2 500	5 600	1.17
8	200:1:8 × 10^{-4}	MMA-*stat*-MAA[e]	CPADB	4	9	ND[f]	ND[f]	20 000	1.21

Note: [a]The polymerizations were performed in the absence of oxygen at room temperature in dimethylsulfoxide (DMSO) using 4.8 W blue LED lamp as a light source (λ_{max} = 461 nm). [b]Monomer conversion was determined by using ^{1}H NMR spectroscopy. [c]Theoretical molecular weight was calculated using the following equation: $M_{n,th} = [M]_0/[RAFT]_0 \times MW^M \times \alpha + MW^{RAFT}$, where $[M]_0$, $[RAFT]_0$, MW^M, α, and MW^{RAFT} correspond to initial monomer concentration, initial RAFT concentration, molar mass of monomer, conversion determined by ^{1}H NMR, and molar mass of RAFT agent. [d]Molecular weight and polydispersity were determined by GPC analysis (DMAC as eluent).[e]$[MMA]_0:[MAA]_0$:[RAFT agent]:[Chl a] = 100:100:1:8 × 10^{-4}. [f]ND: not determined.

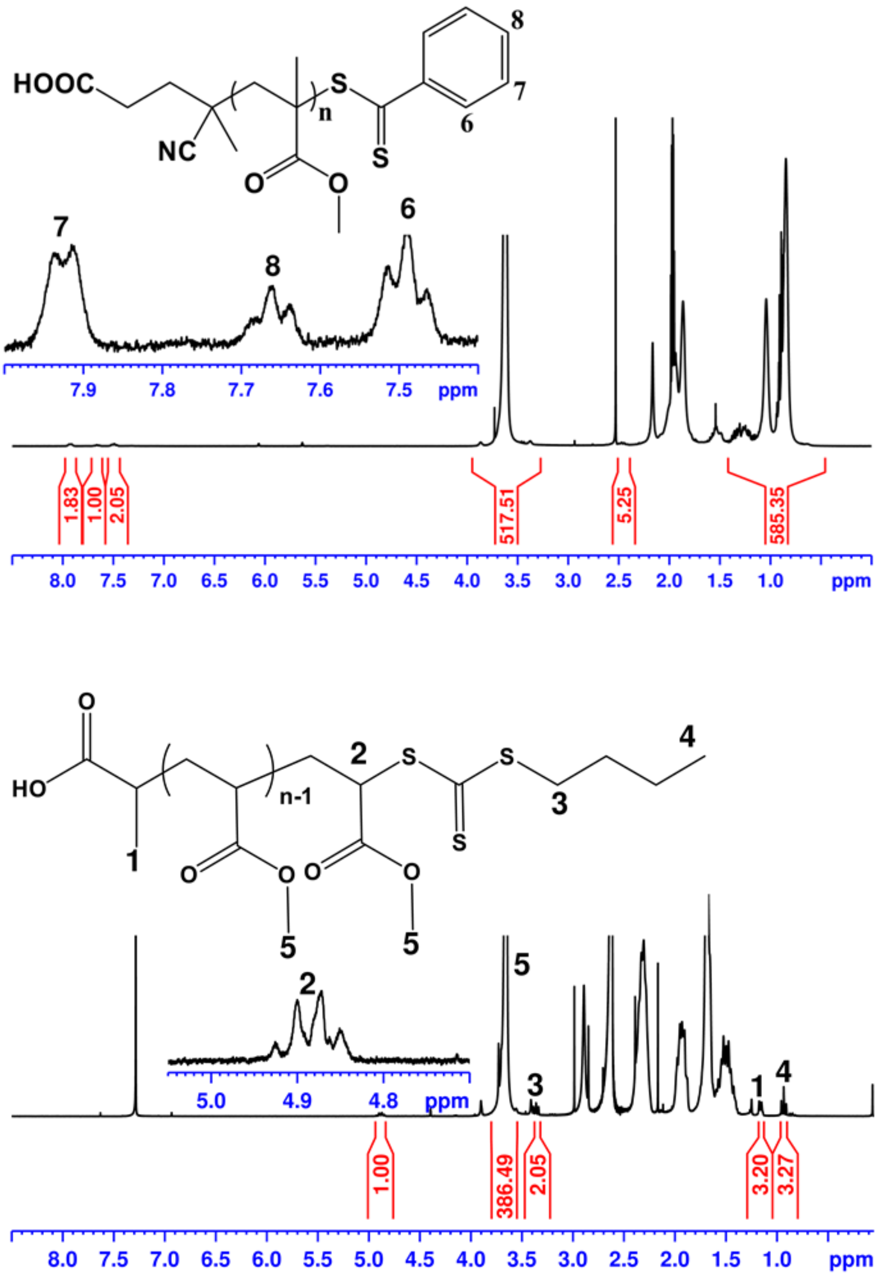

Figure S5. ^{1}H NMR spectra for purified PMMA (top) and PMA (bottom) synthesized under red LED light irradiation ($M_{n,NMR,PMMA}$ = 10 900 g/mol, $M_{n,GPC,PMMA}$ = 11 370 g/mol, $M_{n,theo.,PMMA}$ = 9 700 g/mol, monomer conversion = 47%; $M_{n,NMR,PMA}$ = 10 400 g/mol, $M_{n,GPC,PMA}$ = 8 810 g/mol, $M_{n,theo.,PMA}$ = 8 200 g/mol, monomer conversion = 46%).

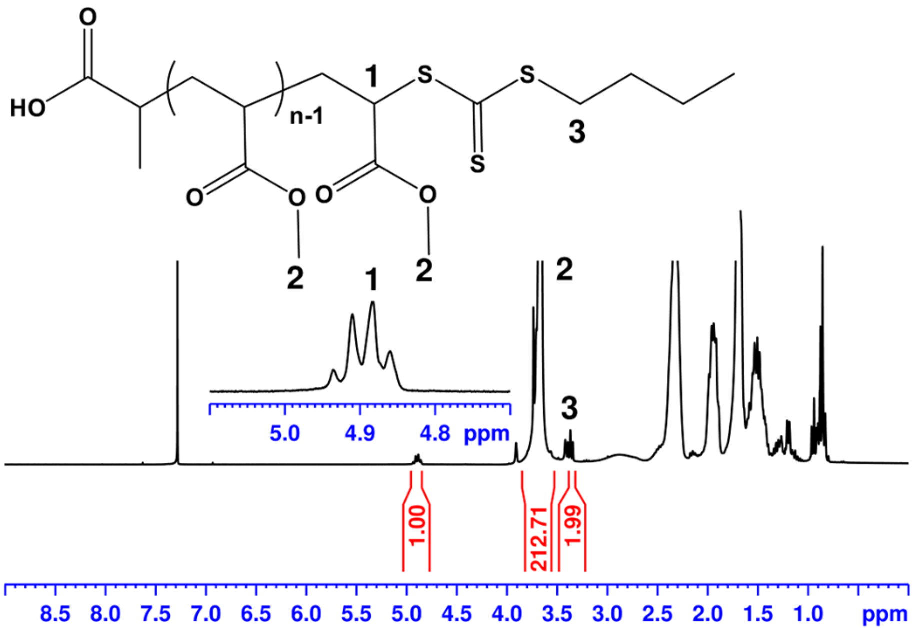

Figure S6. ^{1}H NMR spectra for purified PMA synthesized under blue LED light irradiation ($M_{n,NMR,PMA}$ = 6 400 g/mol, $M_{n,GPC,PMA}$ = 6 580 g/mol, $M_{n,theo.,PMA}$ = 6 900 g/mol, monomer conversion = 39%).

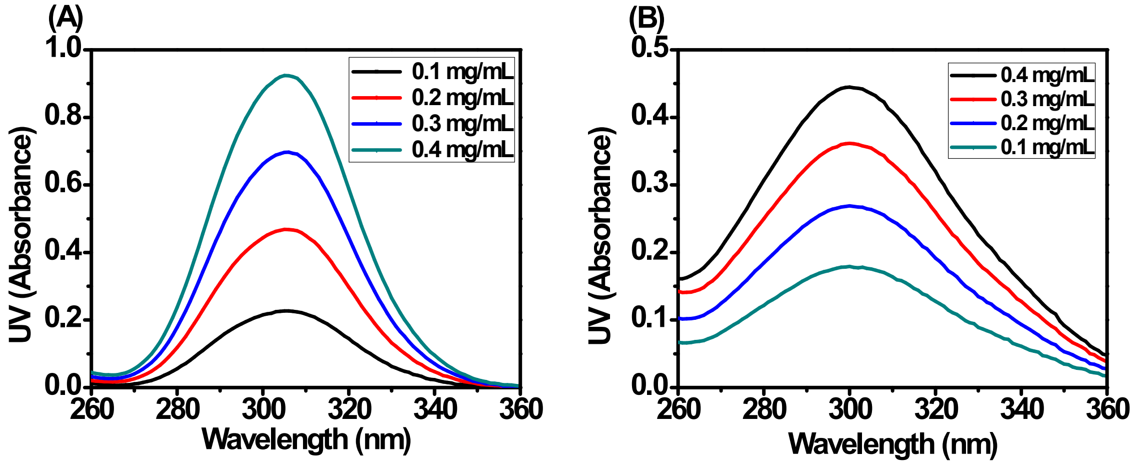

Figure S7. Additional verification of end group fidelity of PMA (A) from Figure S1(B) and PMMA (B) from Figure S1(A) by measuring the absorption of the RAFT end group (BTPA) on the synthesized homopolymers at λ=305 nm using UV-Vis spectrophotometer.

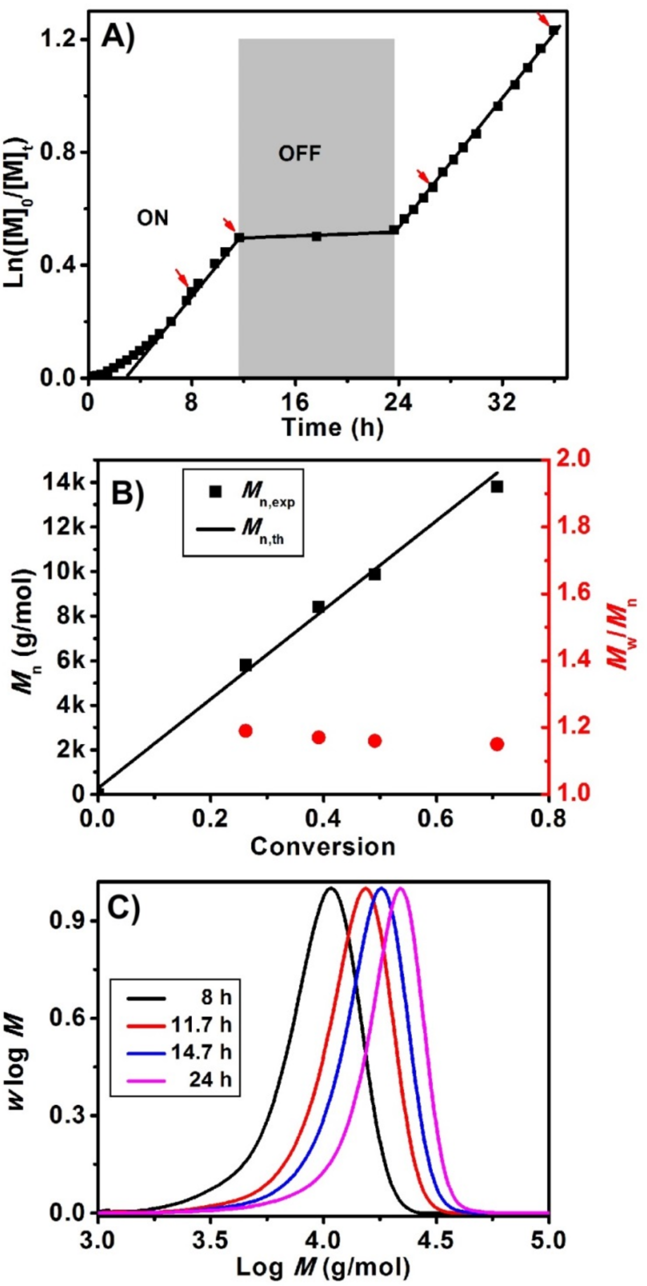

Figure S8. Chl a as a molecular switch for the polymerization of MMA under red light in the absence of oxygen: (A) Plot of $\ln([M]_0/[M]_t)$ against time measured with online Fourier transform near-infrared (FTNIR) spectroscopy for Chl a concentration of 4 ppm (relative to monomer concentration), (B) M_n versus conversion for samples selected at different time points indicated by arrows in A, and (C) molecular weight distributions of PMMA at indicated time points. Arrows indicate aliquots taken from the reaction mixture at specific time points and analyzed by GPC.

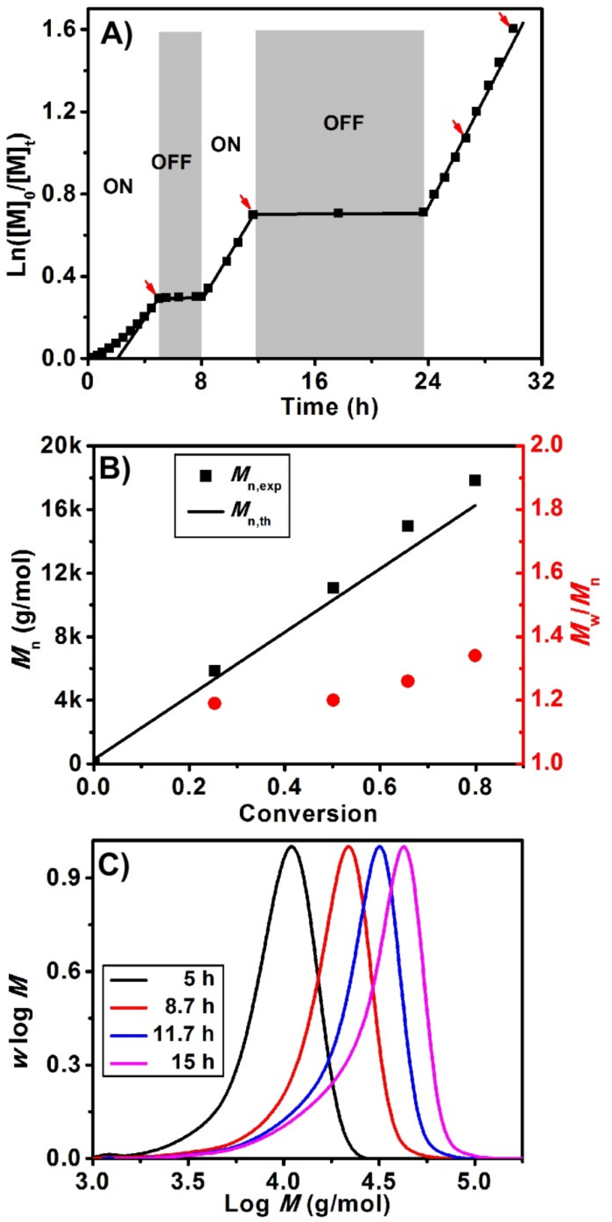

Figure S9. Chl a as a molecular switch for the polymerization of MMA under red light in the absence of oxygen: (A) Plot of $\ln([M]_0/[M]_t)$ against time measured with FTNIR for Chl a concentration of 10 ppm (relative to monomer concentration), (B) M_n versus conversion for samples selected at different time points indicated by arrows in Figure 2 for 10 ppm concentration (relative to monomer concentration), and (C) molecular weight distributions of PMMA at indicated time points. Arrows indicate aliquots taken from the reaction mixture at specific time points and analyzed by GPC.

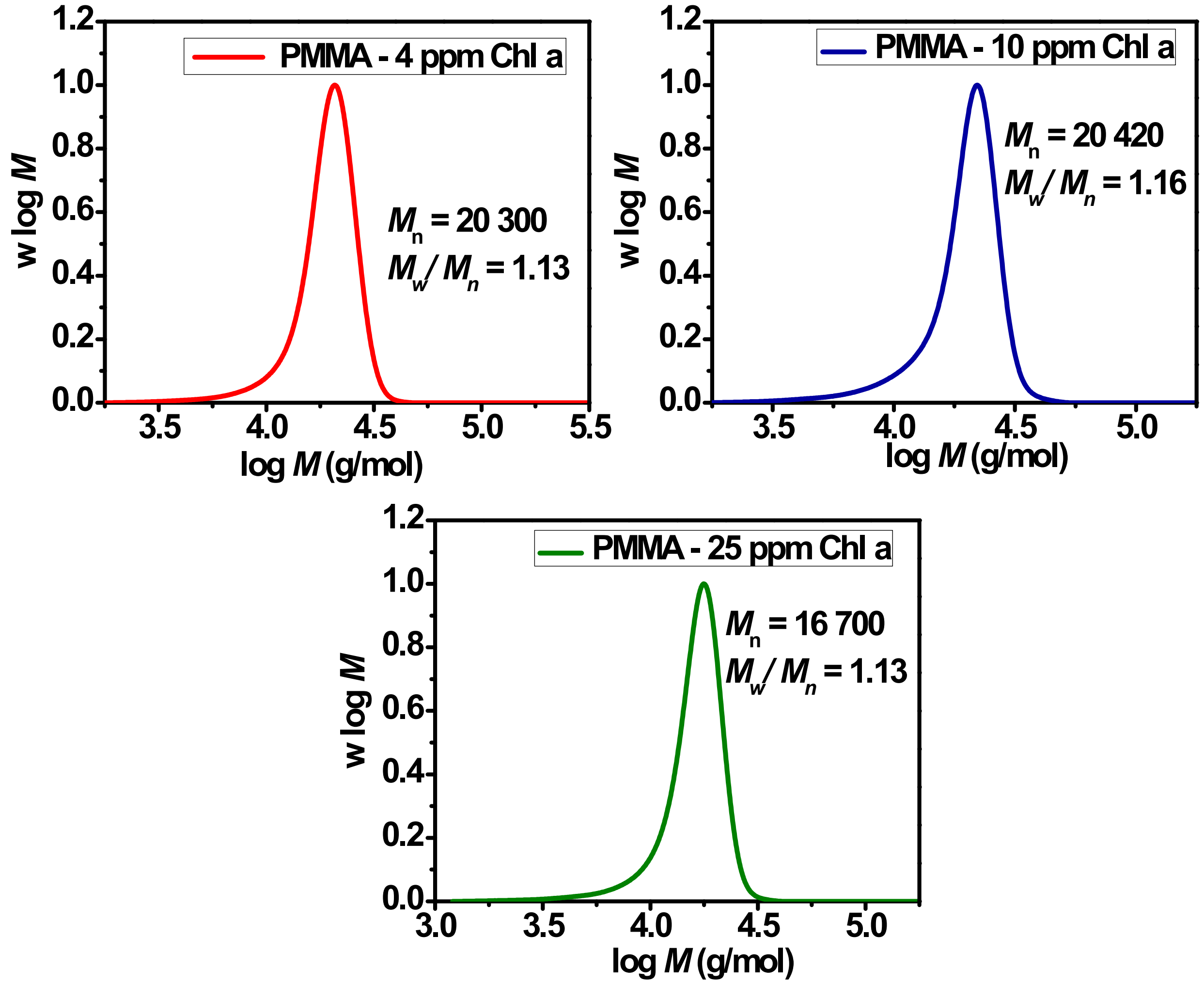

Figure S10. GPC traces for polymerization of MMA ([MMA]:[CPADB]:[Chl a] = 200:1:8 × 10^{-4} (4 ppm)/2 × 10^{-3} (10ppm)/ 5 × 10^{-3} (25 ppm)) under red light in the absence of oxygen with 4 ppm Chl a relative to monomer concentration in 36 hours (top, left), 10 ppm Chl a in 25 hours (top, right) and 25 ppm Chl a in 25 hours (bottom).

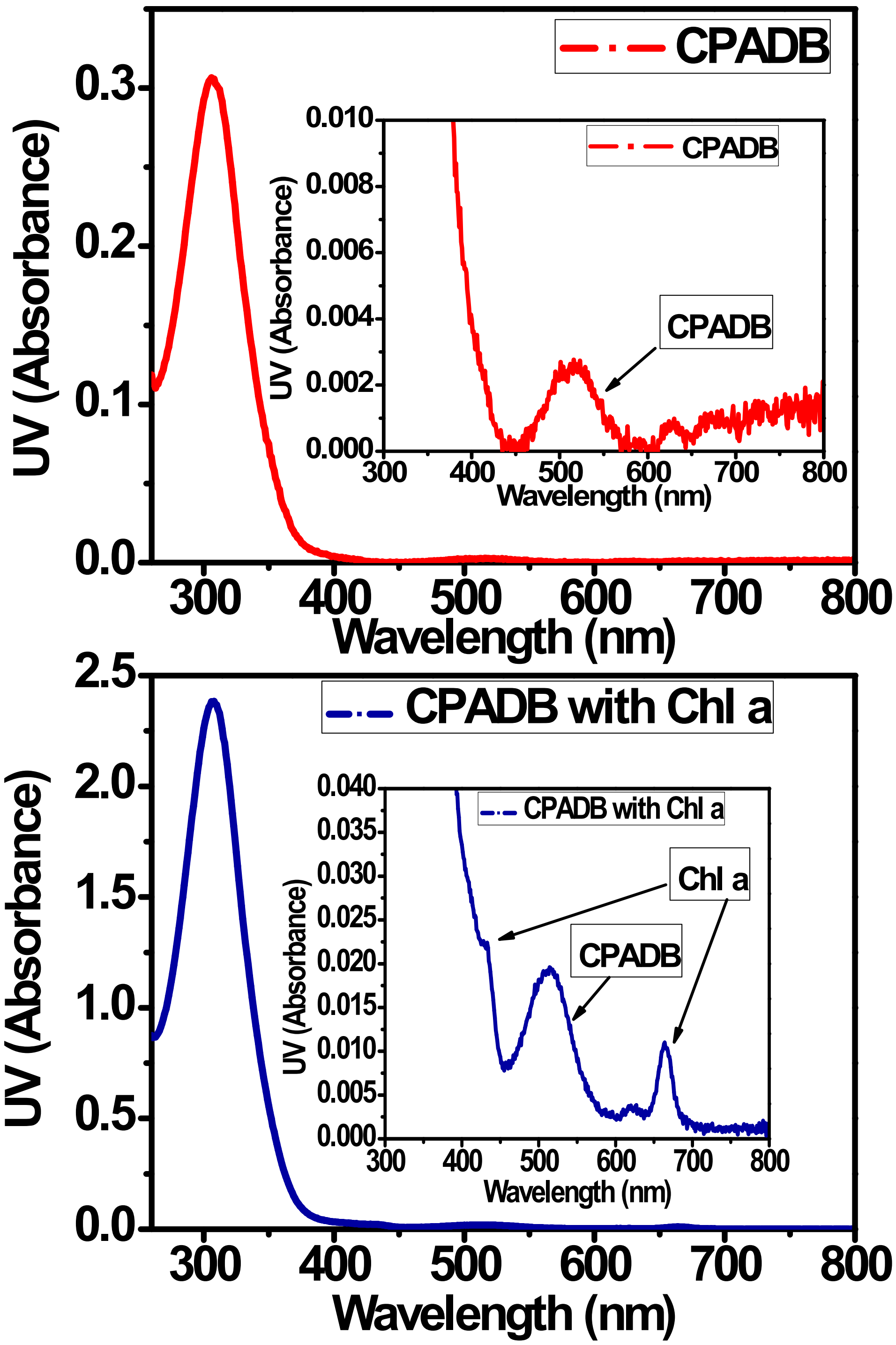

Figure S11A. UV-Vis traces for CPADB (36 μM, top) and CPADB (205 μM, bottom) with Chl a (164 nM, bottom) at a ratio of [CPADB]:[Chl a] = 1: 8 × 10^{-4}.

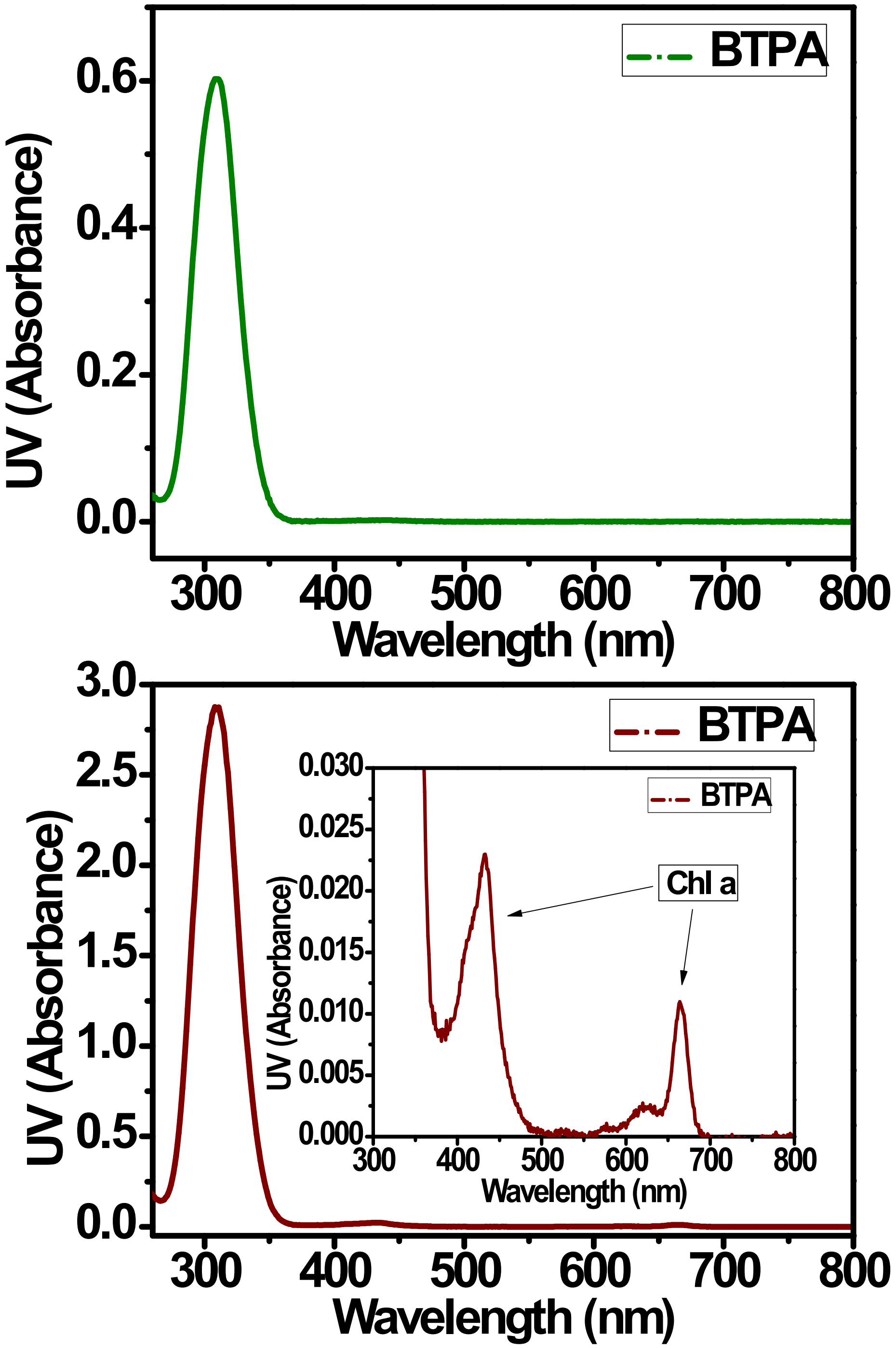

Figure S11B. UV-Vis traces for BTPA (42 μM, top) and BTPA (239 μM, bottom) with Chl a (192 *n*M, bottom) at a ratio of [BTPA]: [Chl a] = 1: 8 × 10^{-4}.

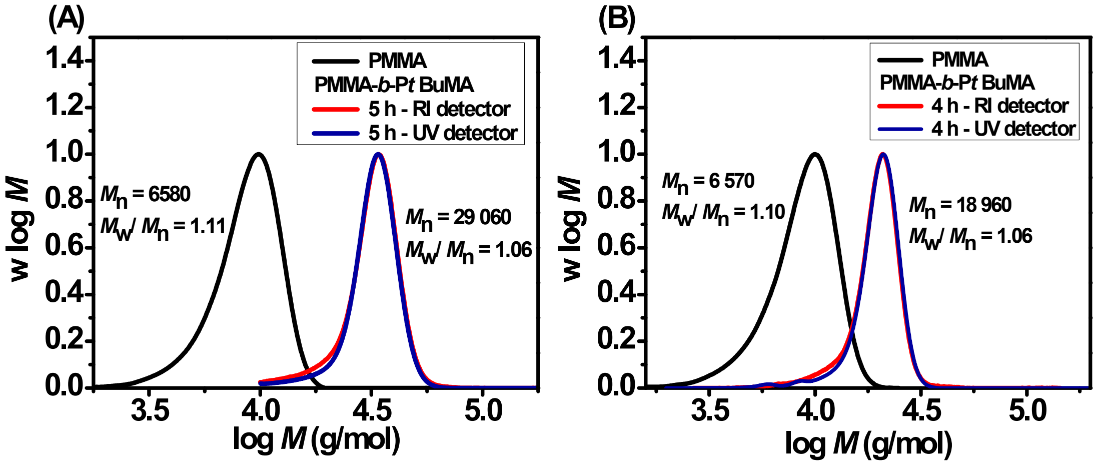

Figure S12. GPC traces for PMMA macroinitiators and their diblock copolymers synthesized with Chl a as the photoredox catalyst and BTPA as the chain transfer agent at room temperature in DMSO ([*t*BuMA]:[macroPMMA]:[Chl a] = 500: 1: 2 × 10^{-3}) : (A) PMMA macroinitiator and PMMA-*b*-P*t*BuMA synthesized under blue light (*t*BuMA conversion: 32%), and (B) PMMA macroinitiator and PMMA-*b*-P*t*BuMA synthesized under red light (*t*BuMA conversion: 17%).

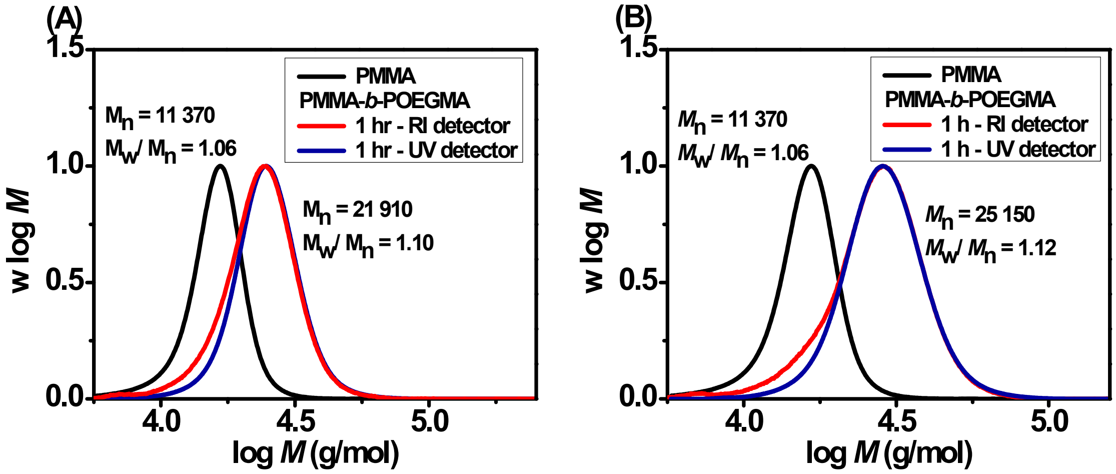

Figure S13. GPC traces for PMMA macroinitiators and their diblock copolymers synthesized with Chl a as the photoredox catalyst and BTPA as the chain transfer agent at room temperature in DMSO ([OEGMA]:[macroPMMA]:[Chl a] = 500: 1: 2 × 10^{-3}): (A) PMMA macroinitiator and PMMA-*b*-POEGMA synthesized under red light (OEGMA conversion: 10%), and (B) PMMA macroinitiator and PMMA-*b*-POEGMA synthesized under blue light (OEGMA conversion: 10%).

References

[1] a) H. T. Quach, R. L. Steeper, G. W. Griffin, *J. Chem. Educ.* **2004**, *81*, 385; b) A. Johnston, J. Scaggs, C. Mallory, A. Haskett, D. Warner, E. Brown, K. Hammond, M. M. McCormick, O. M. McDougal, *J. Chem. Educ.* **2013**, *90*, 796-798.

[2] A. R. Wellburn, *J. Plant Physiol.* **1994**, *144*, 307-313.
